# Exercise induces favorable metabolic changes in white adipose tissue preventing high‐fat diet obesity

**DOI:** 10.14814/phy2.14929

**Published:** 2021-08-17

**Authors:** Babu R. Maharjan, Sergio F. Martinez‐Huenchullan, Susan V. Mclennan, Stephen M. Twigg, Paul F. Williams

**Affiliations:** ^1^ Greg Brown Diabetes & Endocrinology Laboratory Sydney Medical School University of Sydney Sydney Australia; ^2^ Department of Biochemistry Patan Academy of Health Sciences School of Medicine Lalitpur Nepal; ^3^ Faculty of Medicine School of Physical Therapy Universidad Austral de Chile Valdivia Chile; ^4^ New South Wales Health Pathology Sydney Australia; ^5^ Department of Endocrinology Royal Prince Alfred Hospital Sydney Australia

**Keywords:** adipose tissue, exercise, fat depot, high‐fat diet, obesity

## Abstract

Diet and/or exercise are cost effective interventions to treat obesity. However, it is unclear if the type of exercise undertaken can prevent the onset of obesity and if it can act through different effects on fat depots. In this study we did not allow obesity to develop so we commenced the high‐fat diet (HFD) and exercise programs concurrently and investigated the effect of endurance exercise (END) and high‐intensity interval training (HIIT) on changes in cellular adipogenesis, thermogenesis, fibrosis, and inflammatory markers in three different fat depots, on a HFD and a chow diet. This was to assess the effectiveness of exercise to prevent the onset of obesity‐induced changes. Mice fed with chow or HFD (45% kcal fat) were trained and performed either END or HIIT for 10 weeks (3 x 40 min sessions/week). In HFD mice, both exercise programs significantly prevented the increase in body weight (END: 17%, HIIT: 20%), total body fat mass (END: 46%, HIIT: 50%), increased lean mass as a proportion of body weight (Lean mass/BW) by 14%, and improved insulin sensitivity by 22%. Further evidence of the preventative effect of exercise was seen significantly decreased markers for adipogenesis, inflammation, and extracellular matrix accumulation in both subcutaneous adipose tissue (SAT) and epididymal adipose tissue (EPI). In chow, no such marked effects were seen with both the exercise programs on all the three fat depots. This study establishes the beneficial effect of both HIIT and END exercise in preventing metabolic deterioration, collagen deposition, and inflammatory responses in fat depots, resulting in an improved whole body insulin resistance in HFD mice.

## Graphical abstract

Both END and HITT forms of exercise can significantly prevent metabolic deterioration, collagen deposition and decrease inflammatory responses in white fat depots, and improve whole body insulin resistance when started concurrently with mice fed a high fat diet.

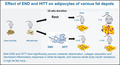

## 1 INTRODUCTION

Obesity and its consequences have become a leading public health challenge worldwide (Kelly et al., 2008; WHO, 2017). Excess adiposity in obesity is an established risk factor in many metabolic diseases including insulin resistance, type 2 diabetes, hypertension, nonalcoholic fatty liver disease, polycystic ovarian disease, and certain cancers (Berrington de Gonzalez et al., 2010; Makki et al., 2013). Therefore, strategies to promote weight loss are encouraged since improvements in the comorbidities of obesity, such as hypertension, dyslipidemia, insulin resistance, and type 2 diabetes have been described (Oster et al., 1999; Santosa et al., 2007; Sjostrom et al., 1999). In that context, lifestyle modifications, such as diet and exercise are effective weight loss interventions in obesity (Cheng et al., 2018; Ma et al., 2017), and exercise can have a beneficial effect irrespective of weight loss (Thyfault & Wright, 2016).

The impact of different exercise types on preventing the changes in adipose tissue subtypes and metabolic health of animals on a HFD is sparse. Our recent study on the effect of constant‐moderate endurance (END) and high‐intensity interval training (HIIT) exercise on skeletal muscle in HFD and chow mice indicated a preventative effect of END which improved skeletal muscle metabolic health and increased the production of muscle adiponectin (Martinez‐Huenchullan et al., 2018). Current studies on adipose tissue show that END, characterized by moderate intensity exercise (40%–70% VO_2_ max) improved insulin sensitivity and delayed the onset of type 2 diabetes (Knowler et al., 2002; Pan et al., 1997). END was able to reduce adipocyte size, inflammation, and collagen deposition in EPI (Linden et al., 2014) and to increase mitochondrial content in SAT (Otero‐Diaz et al., 2018), EPI (Bostrom et al., 2012), and brown adipose tissue (BAT) (Xu et al., 2011) in already obese mice. HIIT which involved brief intermittent bouts of vigorous activity (90%–100% of VO_2_ max) followed by periods of lower activity (40%–70% VO_2,_ or active rest), elicited similar metabolic benefits to END in a shorter time (Cocks et al., 2016; Marcinko et al., 2015; Marquis‐Gravel et al., 2015). HIIT lowered blood glucose and lowered markers of insulin resistance independently of its effects on body mass or adiposity (Cocks et al., 2010; Marquis‐Gravel et al., 2015). In addition, HIIT in obese animals was also associated with a greater reduction in metabolically damaging abdominal fat but was not related to loss of subcutaneous fat (Despres et al., 1988; Tremblay et al., 1990).

Although there is evidence of the effect of END and HIIT as a treatment for obesity in already obese animal models, there is a limited knowledge of their potential to prevent the changes in the different fat depots in preventing weight gain when HFD and exercise were commenced together. In this study, we investigated the ability of END and HIIT to prevent whole body metabolic changes in three adipose tissues depots: subcutaneous, epididymal as a central fat store, and brown adipose tissue (SAT, EPI, and BAT, respectively). Markers of adipogenesis, thermogenesis, inflammation, and fibrosis were examined. We hypothesized that in HIIT as a more intense, vigorous physical activity would be of greater benefit to white and brown adipose tissue metabolism than END, but that a benefit overall would be observed in both exercise regimens.

## 2 MATERIALS AND METHODS

### 2.1 Ethical approval

The study was approved by The University of Sydney Animal Ethics Committee (Protocol#2015/816). The experiments described were carried out according to the guidelines laid down by the New South Wales Animal Research Act and the eighth Edition of the Australian code for the care and use of animals for scientific purposes.

### 2.2 Animal characteristics

In this study, 72 male C57BL/6 J mice were used (Animal Resource Centre). Animals were housed in Charles Perkins Centre (CPC) Laboratory Animal Services of The University of Sydney. Based on dietary and exercise intervention, these animals were randomized into six groups each containing 12 mice (Chow Untrained, Chow+END, Chow+HIIT, HFD Untrained, HFD+END, and HFD+HIIT). Dietary and exercise interventions were commenced at same time and continued for 10 weeks. Animals on chow diet were fed on standard laboratory chow (12% fat) (Meat free mouse diet; Specialty Feeds^®^) and the HFD (45% fat) was prepared in‐house (Lo et al., 2011) and fed ad libitum. Animals were caged in a group of 5–7 in each cage. Mice were placed in a sealed box and euthanized with isoflurane (3%) in oxygen (Stinger^®^, Advanced Anaesthesia Specialist). Blood was collected by cardiac puncture and SAT (from the inguinal region), EPI (from epididymis), BAT (from the intrascapular region), and liver tissue were collected and stored for later analysis.

### 2.3 Animal phenotyping

As described in earlier study (Martinez‐Huenchullan et al., 2018), animal phenotyping and metabolic study was done after 10 weeks of exercise then mice were euthanized after 1 week from last exercise session. Mouse body weights were measured once a week. Spontaneous physical activity and total energy expenditure were determined at the end of the 10‐week program, when individual mice were placed in a Promethion^®^ metabolic cage (Sable Systems International) for ~48 h period with ad libitum access to food and water. This measured their spontaneous physical activity, total energy expenditure, and respiratory quotient (RQ). After a 4‐h period of acclimatization, several metabolic parameters and voluntary running wheel usage were determined and combined to obtain an indication of the total spontaneous physical activity. Echo MRI (EchoMRITM 900 system) was used to measure the body composition (total body fat and lean mass) of animals at the end of the 10‐week programs. Insulin sensitivity was determined using an insulin tolerance test (ITT) as previously described (Lo et al., 2011). Plasma insulin was measured by ELISA (Merck Millipore) according to the manufacturer's instructions.

### 2.4 Exercise programs

As described previously (Martinez‐Huenchullan et al., 2018), acclimation to exercise was performed for 1 week (6 m/min for 10 min) on a treadmill and the maximal running capacity (MRC) was determined by running the mice at 6 m/min and then progressively increasing speed by 3 m/min every 3 min until the mouse was exhausted. Exhaustion was defined by the inability of the animal to reach the end of the lane after being encouraged with five mechanical stimuli delivered with a soft brush within 1 min. The final speed was considered as 100% MRC and used to determine the speed of END and HIIT exercise for both chow and HFD. The exercise programs were END exercise at a constant speed of 17 m/s (70% MRC for 40 min) and for HIIT exercise with eight bouts of vigorous activity [21 m/min (90% MRC) for 2.5 min] interspersed with periods of active rest [12 m/min (50% MRC) for 2.5 min]. The average intensity, exercise time, and distance covered per session was similar between END and HIIT. Exercise was carried out in the morning, three sessions per week for 10 weeks. Animals in the untrained group were not exposed to exercise. Mice refusing to run more than twice in the same week were excluded from the study. This resulted in removal of one animal in each of chow+END, HFD+END, and chow+HIIT groups, and the removal of three in HFD+HIIT groups.

### 2.5 Measurement of gene expression in adipose tissue

In individual adipose tissue depots, gene expression of markers for adipogenesis, thermogenesis, ECM remodeling, inflammation, and tissue insulin resistance were measured by qRT‐PCR. Tissue was homogenized in a Fast prep homogenizer (MP Biomedical). RNA was extracted using RNeasy Lipid Tissue Mini Kit (Qiagen) and was quantitated using the Nanodrop^™^ (Thermo‐Fisher Scientific). RNA quantity and purity was determined by Nanodrop measurement of RNA quantity and purity was determined using the Nanodrop to determine that the optical density 260/280 ratio for all samples was between 1.9 and 2.0. Then RNA (2 μg) was reverse transcribed using 50 pmol of oligo(dT)12–18 (Life Technologies) and 0.4 pmol of random hexamers (Life Technologies). As described previously (Maharjan et al., 2020), real‐time qPCR was performed using the automated pipetting platform Freedom EVO‐2 100 (Tecan Life Science) in a Light cycler 480 (Roche). The mRNA levels of specific species were quantitated using the Delta/Delta method with NoNo used as the reference gene. The qRT‐PCR results were expressed as fold change relative to their respective control. The primers used for qPCR are shown in Table 1.

**TABLE 1:** List of Primers used for measurement of mRNA levels in adipose tissue in the study

Primers	Forward	Reverse
PPARγ	5′‐CTGTCGGTTTCAGAAGTGCCT‐3′	5′‐CCCAAACCTGATGGCATTGTGAGACA‐3′
TLE3	5′‐TTGTCACAGGAGCATCAGCAG‐3′	5′‐CAGATTGGGGAGTCCACGTA‐3′
Adiponectin	5′‐CGACACCAAAAGGGCTCAGG‐3′	5′‐ACGTCATCTTCGGCATGACT‐3′
Leptin	5′‐GCTGCAAGGTGCAAGAAGAAG‐3′	5′‐TAGGACCAAAGCCACAGGAAC‐3′
Resistin	5′‐TTCCTGATGTCGGGGAAGTGA‐3′	5′‐GACCGGAGGACATCAGACATC‐3′
PGCα1	5′‐CTGCGGGATGATGGAGACAG‐3′	5′‐TCGTTCGACCTGCGTAAAGT‐3′
PRDM16	5′‐TGACCATACCCGGAGGCATA‐3′	5′‐CTGACGAGGGTCCTGTGATG‐3′
Tbx15	5′‐TGGCAGAAACAGAACTGGACT‐3′	5′‐CCTTGCTGCTTTTGCATGGT‐3′
UCP1	5′‐CATGGGATCAAACCCCGCTA‐3′	5′‐ATTAGGGGTCGTCCCTTTCC‐3′
TNFα	5′‐GACCCTCACACTCACAAACCA‐3′	5′‐ACAAGGTACAACCCATCGGC‐3′
MCP1	5′‐CACTCACCTGCTGCTACTCA‐3′	5′‐GCTTGGTGACAAAAACTACAGC‐3′
Collagen VI	5′‐GAACTTCCCTGCCAAACAGA‐3′	5′‐CACCTTGTGGAAGTTCTGCTC‐3′
TGFβ1	5′‐ACCGCAACAACGCCATCTAT‐3′	5′‐TGCTTCCCGAATGTCTGACG‐3′
CCN2/CTGF	5′‐GAGTGTGCACTGCCAAAGATG‐3′	5′‐TCCAGGCAAGTGCATTGG T‐3′
TIMP1	5′‐CACAAGTCCCAGAACCGC‐3′	5′‐GGATTCCGTGGCAGGC‐3′
TIMP3	5′‐CTTCTGCAACTCCGACATCGTGAT‐3′	5′‐CAGCAGGTACTGGTACTTGTTGAC‐3′
NoNo	5′‐TGCTCCTGTGCCACCTGGTACTC‐3′	5′‐CCGGAGCTGGACGGTTGAATGC‐3′

### 2.6 Protein quantification

Protein was extracted from 200 mg of snap frozen adipose tissue (SAT and EPI) or 70 mg BAT and mixed with 400 μl of RIPA buffer containing a protease inhibitor cocktail (Cat no. 04693159001 Roche) in a 1.5 ml Eppendorf tube. Tissues were homogenized in Eppendorf tubes manually with a plastic pestle and then incubated for 2 h at 4°C in the cold room. The samples were sonicated at an amplitude 5 in a Missonix sonicator (Misonix) with manual pulsing intermittently 3 times for 3 s each to break up the cell membranes and release the intracellular proteins into solution. Samples were centrifuged at 12,000 ***g*** for 15 min at 4°C (Beckman Coulter) and the supernatant carefully transferred to new 1.5 ml of Eppendorf tubes and stored at −80°C for future analysis. Protein quantification was done using the DC (Detergent compatible) protein assay (Bio‐Rad).

As described previously (Maharjan et al., 2020), equal protein loading of wells was made on gels, and band intensities were normalized to the total protein loaded, which had been determined by the stain free technique (Bio‐Rad^®^). Antibody proteins were diluted 1:500 for UCP1 in SAT and EPI, 1:5,000 for BAT, (Catalog number ab10983, Abcam), 1:500 for PRDM16 (Catalog number ab106410, Abcam), 1:500 for CD45 (Catalog number ab10558, Abcam), and 1:10,000 for secondary antibody labelled with peroxidase (Anti‐rabbit IgG, catalog number S9169; Sigma^®^).

### 2.7 Histology and immunohistochemistry

Tissue sections from the paraffin embedded blocks were used for the histological and immunohistochemical study. H and E staining was done on SAT, EPI, and BAT (Details described in Data S1) for the measurement of adipocyte size. The measurements of adipocyte size in SAT and EPI depots were made after imaging an entire hematoxylin stained section (*n* = 3/group), in an automated slide scanner (Olympus). The imaging software, VS‐DESKTOP Virtual Slide System (Olympus), was used to randomly select four areas across tissue sections and the diameter of 50 cells in each area was determined. For irregularly shaped adipocytes, a line was drawn across the maximum diameter. For Picrosirius Red Staining (PSR), tissues slides were first stained in hematoxylin for 10 min, followed by 3–5 dips in acid alcohol, blueing in Scotts water for 30 s, then stained with Picrosirius Red for 1 h and washed in two changes of acidified water (Junqueira et al., 1979; Puchtler et al., 1973). Immunohistochemical staining of SAT and EPI was done for the collagen after retrieving antigen by heating slides in a microwave oven in Tris‐EDTA buffer pH 9.0 for 10–15 min (detail in Data S1).

### 2.8 Statistical analysis

All the data collected from the study were entered into the Prism Graphpad 7 statistical software for data analysis. To test the effects of HFD and exercise in the different outcomes of interest, two‐way ANOVA with Tukey's multiple comparison test was used. Data were expressed as mean ± SD and a *p* < 0.05 was considered statistically significant.

## 3 RESULTS

### 3.1 Exercise programs prevent the HFD‐induced gain in body weight and fat mass

The HFD for 10 weeks induced significant increases in body weight (BW) with an increase total body fat mass from 3.8 g in chow fed to 16.8 g in HFD. This was reflected in the increases in SAT, EPI, BAT, and liver mass (Table 2). Both forms of exercise (END and HIIT) significantly reduced the total weight gain and increase in white fat depots (Table 2). The percentage of lean mass per total body weight was significantly lower in HFD (58%) compared to chow (83%). Both exercise programs maintained the percent lean mass 72% (Figure 1b and Table 2). A beneficial effect of either exercise program on lean mass was not as obvious in chow‐fed mice (Figure 1b, Table 2). The metabolic cage study showed a difference in the RQ for chow and HFD mice (Table 2) with the RQ for HFD reflecting a mixed diet rather than total lipid usage and the chow RQ being higher indicated higher carbohydrate usage.

**TABLE 2:** The effect of HFD and exercise on anthropometric and insulin sensitivity measurements

	Chow+UNT	Chow+END	Chow+HIIT	HFD+UNT	HFD+END	HFD+HIIT
Body weight (BW) (g)	32.5 ± 1.8	31.0 ± 1.8	30.7 ± 1.2	45.2 ± 2.2*	37.4 ± 2.1* ^,^ ^#^	35.9 ± 2.0* ^,^ ^#^
%Total fat/BW	11.6 ± 3.6	9.4 ± 2.3	7.8 ± 2.4	37.2 ± 3.6*	23.7 ± 6.8* ^,^ ^#^	22.9 ± 7.5* ^,^ ^#^
%SAT/BW	0.9 ± 0.3	0.9 ± 0.2	0.8 ± 0.1	2.7 ± 0.5*	1.8 ± 0.5* ^,^ ^#^	1.8 ± 0.6* ^,^ ^#^
%EPI/BW	2.1 ± 0.6	1.9 ± 0.4	1.7 ± 0.4	5.8 ± 0.9*	4.6 ± 1.1* ^,^ ^#^	4.9 ± 1.3*
%BAT/BW	0.5 ± 0.1	0.5 ± 0.1	0.5 ± 0.1	0.7 ± 0.1*	0.7 ± 0.2*	0.7 ± 0.1
%Liver/BW	4.5 ± 0.4	4.5 ± 0.5	4.7 ± 0.3	5.7 ± 0.7*	4.6 ± 0.6^#^	4.6 ± 0.5^#^
Physical activity (a.u.)	316 ± 75	354 ± 51	323 ± 87	211 ± 59*	308 ± 76	304 ± 77
Energy expenditure (kcal/kg 0.75 × h)	7.2 ± 0.8	6.4 ± 0.4	6.6 ± 0.6	6.0 ± 0.5*	6.8 ± 0.8	6.8 ± 0.4
RQ	0.85 ± 0.01	0.86 ± 0.02	0.84 ± 0.02	0.79 ± 0.03*	0.82 ± 0.03	0.81 ± 0.01
FBG (mmol/L)	7.0 ± 0.8	7.2 ± 1.1	7.5 ± 0.9	10.1 ± 1.9*	7.6 ± 2.0^#^	9.3 ± 1.9*
Insulin (ng/ml)	0.68 ± 0.32	1.18 ± 0.62	0.51 ± 0.18	4.29 ± 2.20*	2.21 ± 1.32^#^	2.50 ± 1.58^#^

Note

Parameters of anthropometric measurements, physical activity, energy expenditure, FBG and non‐fasted insulin level. Data are expressed as mean ± SD. Two‐way ANOVA with Tukey's multiple comparison test was used to compare among the diet and exercise interventions.

Abbreviation: a.u., arbitrary unit; RQ, respiratory quotient.

* *p* < 0.05 chow untrained mice vs. HFD untrained, HFD END and HFD HIIT.

# *p* < 0.05 HFD untrained vs. HFD trained.

**FIGURE 1:**
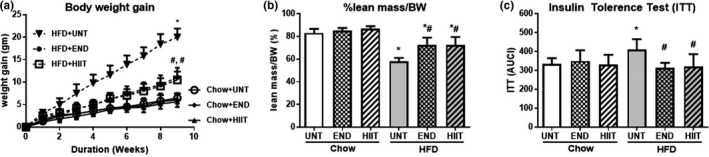
The effect of HFD and exercise on anthropometric and insulin sensitivity measurements. Body weight gain at different time points (a), %lean mass/BW (b) and ITT (c) after the 10 weeks of HFD and exercise. Data are expressed as mean±SD. Two‐way ANOVA with Tukey's multiple comparison test was used to compare among the diet and exercise interventions. *p < 0.05 vs Chow untrained, † vs Chow+END and # vs HFD untrained

### 3.2 Exercise programs enhanced energy expenditure and improved insulin sensitivity

Decreased spontaneous physical activity and total energy expenditure were observed in the HFD mice compared to chow‐fed group (Table 2). Spontaneous physical activity and energy expenditure in chow‐ and HFD‐fed mice were unchanged by exercise (Table 2) and both exercise programs had no effect on FBG or the area under the ITT_(AUC)_ _curve_ in chow‐fed mice (Figure 1c, Table 2). The insulin resistance induced by the HFD was prevented by the exercise programs where a significant reduction in plasma insulin values and a reduced area under the curve of the ITT_(AUC)_ was seen.

### 3.3 Changes in adipogenic markers produced by the HFD and exercise programs

#### 3.3.1 Subcutaneous adipose tissue

**FIGURE 2:**
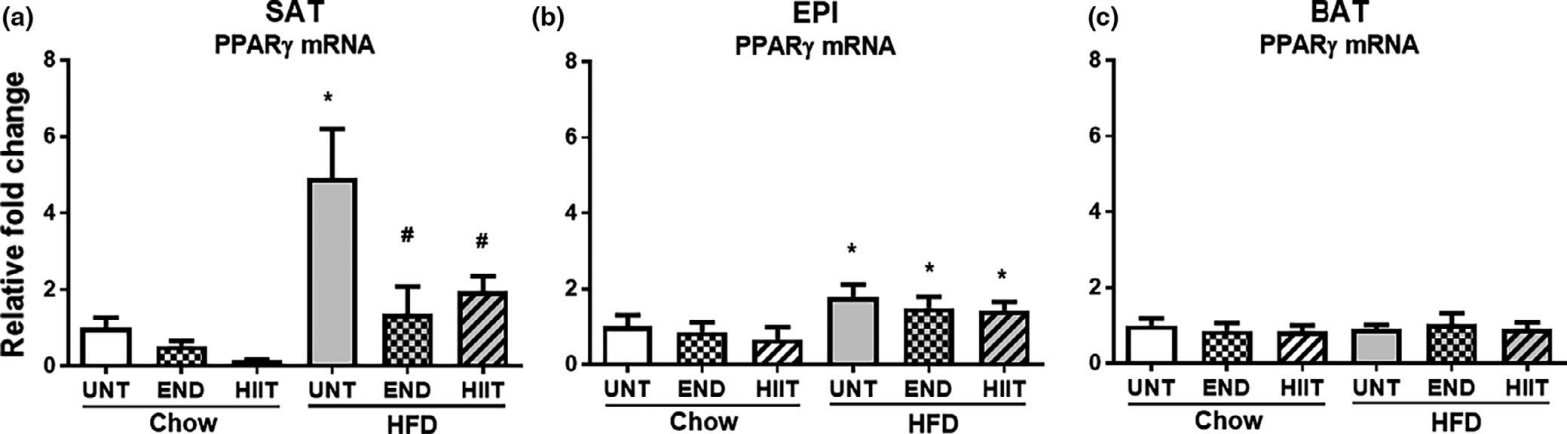
The effect of HFD and exercise on adipogenic markers in SAT, EPI and BAT. PPARγ mRNA levels in SAT (a), EPI (b) and BAT (c). Data are expressed as mean±SD compared with Chow untrained (UNT) after correcting for NoNo as the reference expressed gene. Two‐way ANOVA with Tukey's multiple comparison test was used to compare among the diet and exercise interventions. *p* < 0.05 * vs Chow untrained, † vs Chow+END and # vs HFD untrained

In HFD mice, a significant upregulation in the mRNA of the adipogenic markers in SAT was seen for PPARγ, TLE3, adiponectin, and leptin, which was consistent with the increase in body weight and fat pad mass. In addition, in SAT the size of adipocytes in the HFD cohort (Chow: 46 ± 15 and HFD:80 ± 30 µm, and *p* < 0.05) increased. Each of the exercise programs limited these changes in SAT mRNA (Figure 2, Table 3) and the size of SAT adipocytes (Chow: 46 ± 15; HFD: 80 ± 30, HFD+END: 55 ± 21 and HFD+HIIT: 56 ± 22 µm, and *p* < 0.05) (Figure 3a,b). Chow‐fed mice had no significant change in the adipogenic mRNA markers (Figure 2, Table 3) with both exercise programs but SAT adipocyte size was reduced with HIIT (HIIT: 37 ± 12 µm vs. Chow: 46 ± 15 µm *p* < 0.05). END exercise, in contrast increased SAT adipocyte size compared to untrained controls (Chow: 46 ± 15 µm, END: 62 ± 18 µm, *p* < 0.05) (Figure 3a,b).

**TABLE 3:** The effect of HFD and exercise on adipogenic markers in SAT, EPI and BAT

	(mRNA)	Chow+UNT	Chow+END	Chow+HIIT	HFD+UNT	HFD+END	HFD+HIIT
SAT	TLE3	1.00 ± 0.40	1.51 ± 1.77	Low	2.97 ± 2.20*	0.92 ± 0.58^#^	0.82 ± 0.42^#^
	Adiponectin	1.00 ± 0.40	0.66 ± 0.49	0.09 ± 0.04	5.25 ± 1.84*	0.96 ± 0.61^#^	2.12 ± 0.83^#^
	Leptin	1.00 ± 0.57	0.98 ± 1.14	0.53 ± 0.02	27.45 ± 13.96*	3.46 ± 1.70^#^	3.73 ± 2.15^#^
	Resistin	1.00 ± 0.83	0.74 ± 0.37	0.45 ± 0.32	0.79 ± 0.67	0.71 ± 0.56	0.73 ± 0.68
EPI	TLE3	1.00 ± 0.79	0.91 ± 0.77	0.42 ± 0.40	9.15 ± 1.85*	3.62 ± 1.13* ^,^ ^#^	4.58 ± 1.90* ^,^ ^#^
	Adiponectin	1.00 ± 0.34	0.96 ± 0.32	0.58 ± 0.13	1.65 ± 0.78*	1.47 ± 0.50	1.56 ± 0.36
	Leptin	1.00 ± 0.84	0.85 ± 0.75	0.41 ± 0.40	89.8 ± 32.8*	25.1 ± 10.7* ^,^ ^#^	33.8 ± 18.3* ^,^ ^#^
	Resistin	1.00 ± 0.32	1.08 ± 0.43	0.91 ± 0.32	0.45 ± 0.20*	1.12 ± 0.41^#^	1.19 ± 0.67^#^
BAT	TLE3	1.00 ± 0.19	0.74 ± 0.29	0.54 ± 0.28	1.07 ± 0.30	0.77 ± 0.31	0.88 ± 0.40
	Adiponectin	1.00 ± 0.17	1.13 ± 0.26	1.12 ± 0.16	0.86 ± 0.12	0.90 ± 0.33	0.98 ± 0.14
	Leptin	1.00 ± 0.59	0.59 ± 0.19	0.33 ± 0.23	3.88 ± 1.40*	2.66 ± 1.70	3.05 ± 2.28
	Resistin	1.00 ± 0.29	1.48 ± 0.58	1.44 ± 0.67	0.41 ± 0.27*	0.70 ± 0.39	0.76 ± 0.34

Note

The mRNA levels of other adipogenic markers in SAT, EPI and BAT. Data are expressed as mean ± SD compared to Chow untrained (UNT) after correcting for NoNo as the reference gene. Two‐way ANOVA with Tukey's multiple comparison test was used to compare among the diet and exercise interventions.

*p* < 0.05

* *p* < 0.05 chow untrained mice vs. HFD untrained, HFD END and HFD HIIT.

# *p* < 0.05 HFD untrained vs. HFD trained.

**FIGURE 3:**
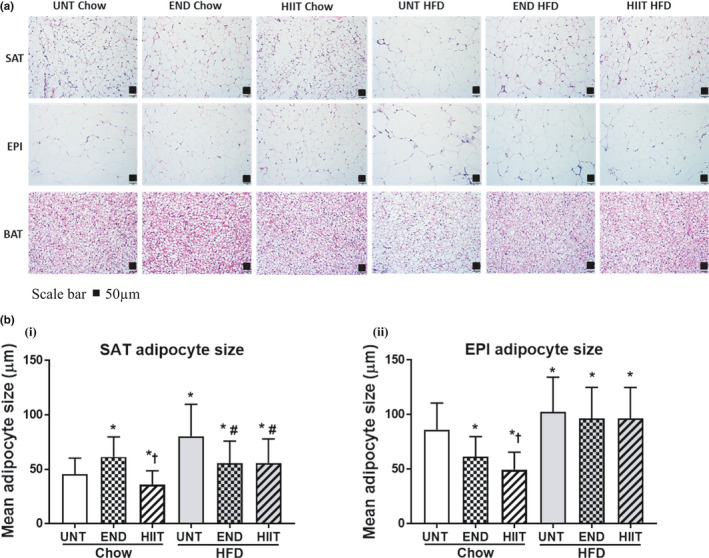
H&E Staining of fat tissues and adipocyte size in SAT and EPI. 3A: H&E Staining for SAT, EPI and BAT after 10 weeks of END and HIIT in chow and HFD (Size bar scale 50 μm). 3B: Histogram showing the measurement of adipocyte size in SAT (i) and EPI (ii) with both dietary and exercise intervention. In‐graph data are expressed in mean±SD. Two‐way ANOVA with Tukey's multiple comparison test was used to compare among the diet and exercise interventions. *p* < 0.05 * vs Chow untrained, † vs Chow+END and # vs HFD untrained

#### 3.3.2 Epididymal adipose tissue

Similar to the mRNA marker changes for adipogenesis seen in SAT, there was a significant upregulation of mRNA levels in EPI for PPARγ, TLE3, adiponectin, and leptin in response to the HFD, and exercise prevented the increases in TLE3 and leptin (Figure 2, Table 3). While both exercise programs limited the increase in EPI adipocytes size (Chow: 86 ± 24, HFD: 102 ± 32, HFD+END: 97 ± 29 and HFD+HIIT: 97 ± 28 µm, and *p* < 0.05) the reduction in size was less than that seen in SAT adipocytes (Figure 3a,b). In chow‐fed mice, neither HIIT, nor END, changed the mRNA of adipogenic markers (Figure 2, Table 3). In chow‐fed animals, the larger adipocyte size of EPI in untrained mice (86 ± 24 µm) was decreased by both HIIT and END, with HIIT appearing to be more effective (HIIT 50 ± 16 µm and END 62 ± 18 µm, and *p* < 0.05) (Figure 3a,b).

#### 3.3.3 Brown adipose tissue

In contrast to the changes seen in white adipocyte markers in response to HFD, only minimal effects were observed in BAT for either HFD or HFD and exercise groups. The exception was in the mRNA for leptin which had increased (Figure 2, Table 3). In chow‐fed mice, both exercise programs had no significant effect on BAT adipogenic markers (Figure 2, Table 3).

### 3.4 The effect of exercise on thermogenic markers

#### 3.4.1 Subcutaneous adipose tissue

**FIGURE 4:**
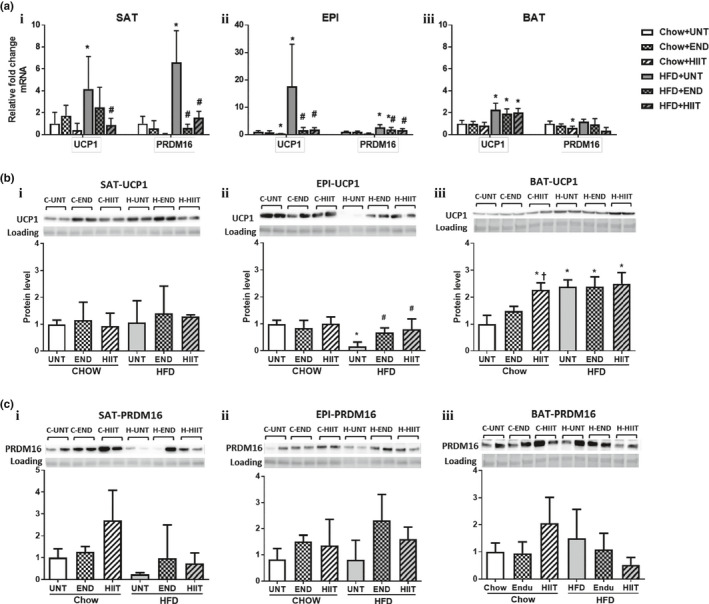
The effect of HFD and exercise on thermogenic markers in SAT, EPI and BAT. (a) mRNA result of UCP1 and PRDM16 in SAT (i), EPI (ii) and BAT (iii) corrected for NoNo as the reference gene. (b) A representative Western blot of UCP1 and a histogram for Protein analysis in SAT (i), EPI (ii) and BAT (iii) (for each group *n* = 4). (c) A representative Western blot of PRDM16 and histogram for Protein analysis in SAT (i), EPI (ii) and BAT (iii) (for each group *n* = 4). In all figures data are expressed in mean ± SD relative to Untrained (UNT) Chow fed animals. Two‐way ANOVA with Tukey's multiple comparison test was used to compare among the diet and exercise interventions. *p* < 0.05 * vs UNT Chow, † vs Chow+END and # vs UNT HFD. UCP1 protein band is detected at 33 kDa and PRDM16 protein at 140 kDa

Both of the exercise programs significantly prevented the HFD‐induced increases in SAT UCP1, PGC1α, PRDM16, and Tbx15 mRNA (Figure 4a and Table 4). While there were significant changes in the mRNA, there were no significant changes in the protein levels of UCP1 or PRDM16 (Figure 4b,c). In chow‐fed mice, both exercise programs had no significant effect on thermogenic markers (Table 4, Figure 4a–c).

**TABLE 4:** The effect of HFD and exercise on thermogenic markers in SAT, EPI and BAT

	(mRNA)	Chow+UNT	Chow+END	Chow+HIIT	HFD+UNT	HFD+END	HFD+HIIT
SAT	PGC1α	1.00 ± 0.63	0.89 ± 0.88	0.063 ± 0.05	3.77 ± 1.39*	1.30 ± 0.95^#^	1.29 ± 0.48^#^
	Tbx15	1.00 ± 0.58	0.93 ± 0.57	0.76 ± 0.45	4.71 ± 2.88*	1.54 ± 1.27^#^	1.79 ± 1.10^#^
EPI	PGC1α	1.00 ± 0.31	0.72 ± 0.14	0.42 ± 0.14*	1.42 ± 0.40	0.97 ± 0.38^#^	1.02 ± 0.50
	Tbx15	1.00 ± 0.63	0.82 ± 0.30	1.21 ± 0.91	3.39 ± 1.03*	1.71 ± 1.19^#^	1.42 ± 0.55^#^
BAT	PGC1α	1.00 ± 0.39	0.89 ± 0.38	1.00 ± 0.57	1.32 ± 0.36	1.12 ± 0.39	1.00 ± 0.19
	Tbx15	1.00 ± 0.91	0.39 ± 0.16	1.07 ± 0.65	0.72 ± 0.49	0.90 ± 0.64	0.45 ± 0.36

Note

The mRNA levels of PGC1α and Tbx15 are shown expressed as mean ± SD compared to Chow untrained (UNT) after correcting for NoNo as reference gene. Two‐way ANOVA with Tukey's multiple comparison test was used to compare among the diet and exercise interventions.

*p* < 0.05

* vs Chow untrained,

† vs Chow+END and

# vs HFD untrained.

#### 3.4.2 Epididymal adipose tissue

In EPI, the mRNA levels for UCP1, PRDM16, and Tbx15 were each significantly increased by the HFD and these changes were prevented by both of the exercise programs (Figure 4a, Table 4). In contrast both programs significantly prevented the decrease in UCP1 protein seen in the HFD mice (Figure 4b). In addition there was a trend for the END but not HIIT to prevent the HFD decrease in PRDM16 protein (*p* = 0.08) (Figure 4c). In chow‐fed mice, both the exercise programs had no significant effect on thermogenic markers (Table 4, Figure 4a–c).

#### 3.4.3 Brown adipose tissue

In BAT, HFD significantly increased both the UCP1 protein and the mRNA but did not change either the mRNA or protein for PRDM16 or the mRNA of the thermogenic markers PGC1α and Tbx15. Both exercise programs had no effect on the HFD‐induced changes in UCP1 and PRDM16 mRNA and protein, or in the gene expression of any of the other thermogenic markers (Table 4, Figure 4a–c). In chow‐fed mice, HIIT exercise significantly increased UCP1 protein, but these occurred in the absence of any changes in the mRNA expression of the thermogenic markers (Table 4, Figure 4a–c).

### 3.5 Exercise prevented HFD ECM accumulation

#### 3.5.1 Subcutaneous adipose tissue

**FIGURE 5:**
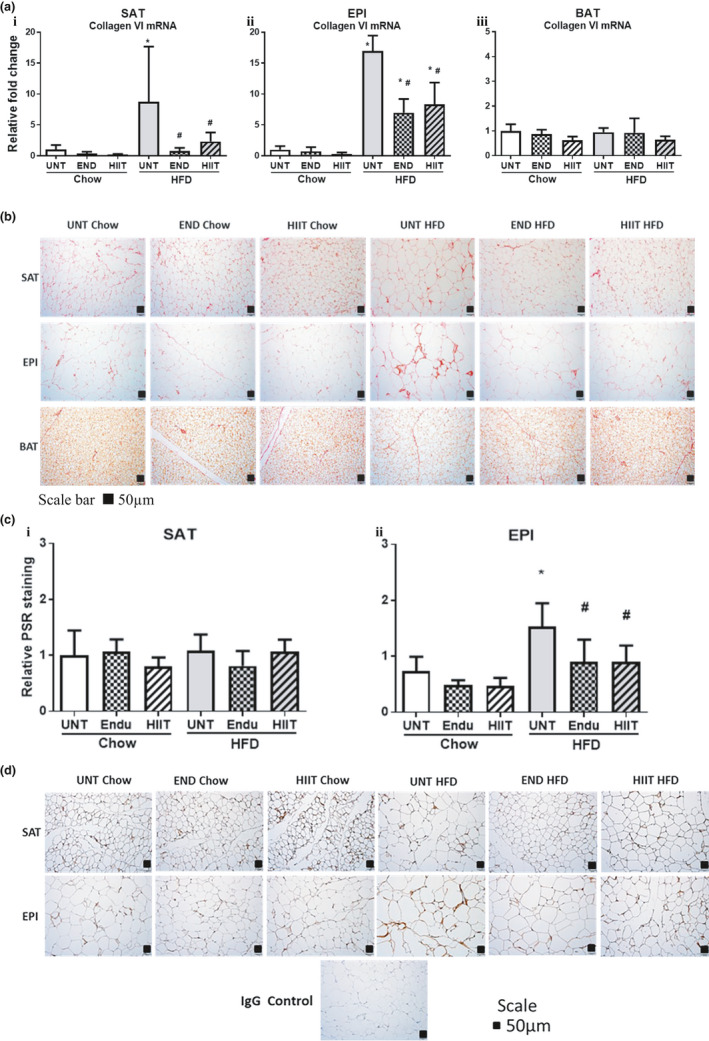
The effect of HFD and exercise on collagen in SAT, EPI and BAT. (a) Histogram for collagen VI mRNA levels in SAT (i), EPI (ii) and BAT (iii) corrected for NoNo as reference gene. (b) A representative PSR Staining for SAT, EPI and BAT (for each group *n* = 5, Scale bar 50 μm). (c) Histogram shows the quantification of PSR staining for SAT (i) and EPI (ii). (d) A representative IHC staining for SAT and EPI (for each group *n* = 3, Scale bar 50 μm). Data are expressed in mean±SD relative to untrained (UNT) Chow. Two‐way ANOVA with Tukey's multiple comparison test was used to compare among the diet and exercise interventions. *p* < 0.05 * vs UNT Chow, † vs Chow+END and # vs UNT HFD

In SAT, HFD increased the mRNA levels for collagen VI and its regulators TGFβ1, CCN2/CTGF, TIMP1, and TIMP3 (Figure 5a, Table 5). Despite the changes in the mRNA levels of these fibrotic markers, we were unable to detect an increase in collagen protein by either Western blot (data not shown), IHC or PSR staining (Figure 5b–d). Once again exercise significantly attenuated the HFD increase in the mRNA levels of collagen VI and the fibrotic markers TGFβ1, CCN2/CTGF, TIMP1, and TIMP3 (Figure 5a, Table 5) but did not change collagen protein levels (data not shown). In chow‐fed mice no significant changes in ECM accumulation markers was observed (Figure 5a, Table 5).

**TABLE 5:** The effect of HFD and exercise on the regulators of collagen in SAT, EPI and BAT

	(mRNA)	Chow+UNT	Chow+END	Chow+HIIT	HFD+UNT	HFD+END	HFD+HIIT
SAT	TGFβ1	1.00 ± 0.55	0.54 ± 0.70	0.05 ± 0.02	16.03 ± 18.26*	0.72 ± 0.51^#^	3.77 ± 5.24
	CCN2	1.00 ± 0.70	1.11 ± 0.76	1.60 ± 0.96	13.93 ± 13.21*	0.61 ± 0.30^#^	2.5 ± 1.77^#^
	TIMP1	1.00 ± 0.40	0.55 ± 0.28	0.15 ± 0.21	3.39 ± 1.10*	0.73 ± 0.26^#^	1.74 ± 1.04^#^
	TIMP3	1.00 ± 0.80	0.66 ± 0.44	0.13 ± 0.05	10.63 ± 8.84*	0.73 ± 0.68^#^	1.28 ± 0.61^#^
EPI	TGFβ1	1.00 ± 0.49	0.79 ± 0.45	0.30 ± 0.21	10.36 ± 5.41*	2.49 ± 0.63^#^	3.13 ± 0.95^#^
	CCN2	1.00 ± 0.62	0.81 ± 0.62	0.18 ± 0.14	10.03 ± 3.47*	3.76 ± 2.11* ^,^ ^#^	4.14 ± 1.93* ^,^ ^#^
	TIMP1	1.00 ± 0.52	0.66 ± 0.42	0.47 ± 0.22	20.92 ± 12.40*	4.41 ± 3.56^#^	4.02 ± 1.31^#^
	TIMP3	1.00 ± 0.79	0.72 ± 0.78	0.26 ± 0.25	5.67 ± 2.10*	3.43 ± 1.12* ^,^ ^#^	3.45 ± 1.30* ^,^ ^#^
BAT	TGFβ1	1.00 ± 0.33	0.80 ± 0.27	0.66 ± 0.21	1.18 ± 0.58	1.19 ± 0.75	0.99 ± 0.79
	CCN2	1.00 ± 0.49	0.84 ± 0.37	0.80 ± 0.41	3.16 ± 1.90*	2.37 ± 1.54	1.74 ± 1.46
	TIMP1	1.00 ± 0.50	0.90 ± 0.57	1.22 ± 0.77	1.60 ± 0.53	0.84 ± 0.19^#^	0.63 ± 0.10^#^
	TIMP3	1.00 ± 0.34	0.63 ± 0.20	0.53 ± 0.26	1.08 ± 0.32	0.75 ± 0.43	0.66 ± 0.31

Note

In each fat depot, mRNA levels of TGFβ1, CCN2/CTGF, TIMP1 and TIMP3 were measured and data are expressed as mean ± SD compared to Chow untrained (UNT) after correcting for NoNo as the reference gene. Two‐way ANOVA with Tukey's multiple comparison test was used to compare among the diet and exercise interventions.

*p* < 0.05

* vs Chow untrained,

† vs Chow+END and

# vs HFD untrained.

#### 3.5.2 Epididymal adipose tissue

The results in EPI contrasted with those in SAT, where not only collagen VI mRNA but also collagen protein accumulation (measured by IHC and PSR staining) was increased in response to the HFD (Figure 5a–d). The finding of increased collagen was reinforced by the concomitant upregulation of the mRNA for the profibrotic factors TGFβ1, CCN2/CTGF, TIMP1, and TIMP3 (Table 5). In this fat depot both of the exercise programs significantly prevented the increase in these mRNA changes and in the histological changes of collagen protein (Table 5, Figure 5a–d). Despite the clear IHC evidence supporting the preventative effect of exercise on the HFD‐induced change in collagen metabolism, we were unable to detect changes in collagen VI protein by Western blotting (data not shown). In the chow‐fed mice, markers of ECM accumulation were not significantly changed with both exercise programs (Figure 5a–d and Table 5).

#### 3.5.3 Brown adipose tissue

In contrast to the changes seen in both of the white fat depots, BAT ECM changes were not seen either in the HFD or in its exercise cohorts. Changes were not detected in the PSR staining for total collagen (Figure 5b) and only minimal changes for gene expression of collagen VI and ECM accumulation markers TGFβ1 and CCN2/CTGF (Figure 5a, Table 5) were seen. There was an increase in the mRNA for the metalloproteinase inhibitor TIMP1 induced by the HFD, which was prevented by both of the exercise programs (Figure 5a, Table 5). In chow‐fed mice mRNA levels of ECM accumulation markers were not significantly different with exercise programs and PSR staining for total collagen remained unchanged (Figure 5b and Table 5).

### 3.6 Exercise‐induced changes in inflammatory markers.

#### 3.6.1 Subcutaneous adipose tissue

**FIGURE 6:**
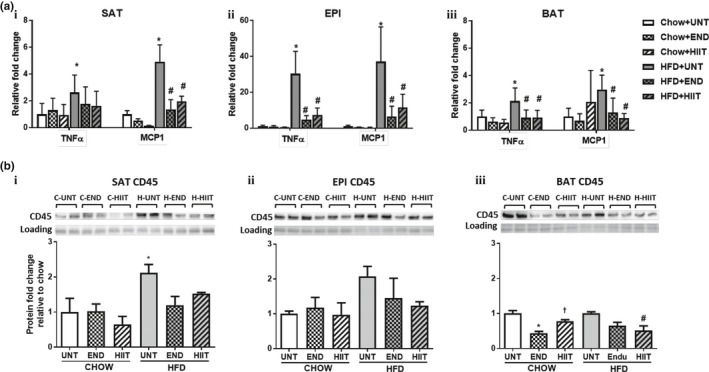
The effect of HFD and exercise on the inflammatory markers in SAT, EPI and BAT. (a) mRNA levels of TNFα and MCP1 in SAT (i), EPI (ii) and BAT (iii) corrected for NoNo as reference gene. In (b) A representative Western blot for CD45 in SAT (i), EPI (ii) and BAT (iii) [Chow represented as (C) and HFD as (H)] and quantification (for each group *n* = 4). In both figures data are mean ± SD relative to Chow untrained (UNT). Two‐way ANOVA with Tukey's multiple comparison test was used to compare among the diet and exercise interventions. *p* < 0.05 * vs UNT Chow, † vs Chow+END and # vs UNT HFD. CD45 protein band is detected at 140 kDa

In SAT, both of the exercise programs prevented the HFD‐induced increase in inflammatory mRNA markers (TNFα, MCP1) and CD45 protein (Figure 6a,b). But in chow‐fed mice, a significant change in inflammatory markers was not observed with both the exercise programs (Figure 6a,b).

#### 3.6.2 Epididymal adipose tissue

Similar to the changes in SAT, in EPI fat pads, both of the exercise programs were able to prevent the HFD‐induced increase in the mRNA levels of TNFα and MCP1 but the decrease in CD45 protein failed to reach significance from the HFD‐induced increase (Figure 6a,b). In chow‐fed mice, both the exercise programs did not have significant effect on inflammatory marker (Figure 6a,b).

#### 3.6.3 Brown adipose tissue

In BAT, similar changes in inflammatory markers to those seen in the white fat depots were observed in response to the HFD. Both of the exercise programs significantly prevented the HFD increase in the mRNA levels of TNFα and MCP1 (Figure 6a), but only HIIT was able to alter the increased CD45 protein of the HFD (Figure 6b). The exercise programs did not alter the inflammatory marker mRNA levels in BAT from chow‐fed mice (Figure 6a,b).

## 4 DISCUSSION

The current study demonstrated that when commenced concurrently both exercise programs significantly decreased the HFD increase in weight gain and insulin resistance (Fisher et al., 2015; Jelleyman et al., 2015; Linden et al., 2014). Despite exercise improving insulin sensitivity in the HFD cohort, only END lowered the blood glucose to the levels seen in chow‐fed mice (Table 2). The difference in diets was reflected in the RQ where chow‐fed animals had an RQ of 0.85 and HFD mice had an RQ of 0.79 looked to be reflecting a mixed diet response. As HIIT is known to increase adrenergic activity and exercise‐induced uptake it is possible that increased catecholamine levels maintained the higher blood glucose as insulin levels while lower than those in the HFD were not different in either of the exercise groups (Adams, 2013; Guelfi et al., 2005). Since FBG was measured 1 week after the cessation of exercise, any increase in the catecholamine levels would have to have been a chronic response to exercise. In type 1 diabetes, continuous moderate intensity exercise (like END) reduced the blood glucose more than short bursts of intense exercise (like HIIT) (Guelfi et al., 2005).

As would be expected, in chow‐fed mice in the absence of excess calories, both exercise programs would not affect FBG and ITT_(AUC)_ levels. This is consistent with other studies of moderate intensity (Owens et al., 1977) and also for HIIT exercise programs (Jelleyman et al., 2015). Babraj et al. (2009) and Richards et al. (2010) did not demonstrate a difference in the FBG of healthy individuals; however, they did find that HIIT exercise caused an improvement in insulin sensitivity.

Improved insulin sensitivity has been associated with decreased fat mass (Linden et al., 2014) and reduced adipocyte size (Craig et al., 1981; Shadid & Jensen, 2006) and we found that exercise prevented these changes in HFD mice. The significance of our study was that in HFD (i) each of the exercise programs were beneficial in preventing a gain in total fat, SAT, liver, and EPI mass and in maintaining adipocyte size of the different fat pads to levels similar to those seen in untrained chow mice; (ii) each of the exercise programs significantly prevented the HFD‐induced adipogenesis, but the preventive effect was more pronounced in SAT than in EPI. This was shown by the markedly lower levels of PPARγ, TLE3, adiponectin, and leptin mRNA in SAT and minimally reduced levels of TLE3 and leptin mRNA in EPI. In that context, Vieira et al. (2009) have also shown decreased leptin mRNA in EPI after an endurance exercise program.

Our study also showed that the effect of HFD to increase fat pad mass and fat cell size as hyperplastic and hypertrophic changes in adipose tissue, was coincident with the development of insulin resistance. Both exercise programs prevented the development of insulin resistance and the hyperplastic and hypertrophic changes in SAT and EPI contributing to a better metabolic state in HFD‐fed exercised mice. An important observation in our study was that exercise produced a greater reduction in the adipogenic markers and adipocyte size in SAT than it did in EPI which highlights that the increased metabolic adaptation of SAT is an important factor in the body's response to extra calories.

We found a relative decrease in the lean mass of HFD‐fed animals and both exercise programs preserved lean mass. These findings are consistent with the observation of Villareal et al. (2004), and other studies with similar exercise programs to our END and HIIT that showed increased lean mass (Pavlou et al., 1985; Peterson et al., 2011) associated with an improvement in insulin resistance (Brochu et al., 2008; Fukushima et al., 2016; Kawanaka, 2011; Koivisto et al., 1986). Using the parameters derived from the metabolic cage studies we did show that both END and HIIT prevented the decrease in spontaneous physical activity and energy expenditure seen in the HFD, which may be an important contributor in controlling weight gain that otherwise occurs without exercise. This concept was consistent with the study done by Wu et al where they showed similar increases in energy expenditure and spontaneous physical activity after exercise during the dark cycle (endurance 75%–85% VO2 5 days/week for 8 weeks) in HFD with established obesity and in chow rat (Wu et al., 2014).

It is evident from published literature in various in vivo models that SAT has a greater potential to become multilocular and to increase UCP1 (or undergo beiging) compared with epididymal WAT (Cinti, 2011, 2012), and that exercise had the ability to increase UCP1 levels in SAT more so than in EPI (Bostrom et al., 2012). In this report we now show that both of the exercise programs prevented the HFD‐ associated decrease in UCP1 and PRDM16 proteins in EPI. This is supported by the mRNA changes in EPI where PRDM16 and PPARγ remained high in exercising HFD mice. In contrast, UCP1 protein remained unchanged by HFD or exercise in SAT, and PRDM16 which was reduced by HFD, approached chow levels with exercise (Figure 4b,c). EPI changes reflect a positive effect of exercise on UCP1 in restoring normality, which was observed previously by Xu et al. (2011). What is new and exciting in our study is that UCP1 protein levels remained stably elevated for 1 week after the cessation of exercise, indicating a beneficial memory effect of exercise training, where cellular adaptation to training can continue even after exercise discontinued a week earlier. Other cellular adaptations occurred in EPI where by preventing the decrease in UCP1, exercise may preserve the beiging of adipocytes. However, changes in the mRNA levels of EPI for some thermogenic markers (UCP1, PRDM16, and Tbx15) were counterintuitive because the HFD increased their mRNA levels and decreased UCP1 protein and both of the exercise programs prevented the mRNA changes and the decrease in UCP1 protein. The discordance between protein and mRNA levels in HFD could be a compensatory mechanism where increased mRNA occurred in response to decreased UCP1 protein levels. Also, the effect of exercise lowering UCP1 mRNA in HFD‐fed mice may have reverted by the time of tissue harvest which was 1 week after the cessation of exercise (Ringholm et al., 2013) but the protein levels remained high due to a longer half‐life of proteins than mRNA tissue levels. The consistent increase in UCP1 mRNA and protein levels in BAT of HFD‐fed animals indicated that BAT was increasing thermogenesis in an attempt to burn excess calories and again the effect of exercise maintained UCP1 protein levels in BAT after exercise had ceased (Figure 4b). The increased PGC1α may help to stabilize the PRDM16 and this would be preventative of fibrosis (reduced collagen in SAT, Figure 5) and maintain the production of fat cells from progenitors in SAT but not EPI (Hasegawa et al., 2018).

In chow‐fed mice, there was no significant change in UCP1 and PRDM16 in all the three fat depots either with END or HIIT (Figure 4a–c), which contrasts with a study reported by Stanford et al where there was an increase in UCP1 mRNA (Stanford et al., 2015) and PRDM16 protein (Vidal & Stanford, 2020) in animals with established obesity before exercise. Moreover, recent studies at thermoneutral conditions examining effects of exercise on thermogenesis and mitochondrial activity in WAT (McKie et al., 2019; Raun et al., 2020) and BAT (Aldiss et al., 2020) did not show a significant effect. In contrast a study conducted at a lower laboratory temperature found the effect of exercise on these parameters to be variable (Vidal & Stanford, 2020). Since animals in our study were caged at 22 ± 2℃, an increased BAT UCP1 was expected (Hirata, 1982) and in our case there was an increase in UCP1 protein with HIIT but not so with END. This difference in UCP1 between END and HIIT thus reflects differential effects of these exercise programs in different tissues.

It has been proposed that END exercise increases UCP1 in peripheral SAT rather than in core BAT by promoting peripheral fat burning and favoring dissipation of heat without affecting core body temperature (Sepa‐Kishi & Ceddia, 2016). In our study, we did not observe any differential regulation of thermogenic markers in response to the HFD in SAT and BAT with either of the exercise programs. In contrast in chow‐fed mice, though END had no effect, HIIT exercise increased UCP1 in BAT but not in SAT. This highlighted different responses to the forms of exercise examined in our study.

In terms of matrix changes in adipose tissue, each exercise program prevented HFD‐induced collagen accumulation in EPI as shown by changes in gene expression, PSR staining and IHC staining, all of which is consistent with earlier observations (Kawanishi et al., 2013). The scattered nature of the collagen accumulation observed in the tissue sections by PSR and IHC staining tissues may account for the inconsistent quantification of collagen VI protein by the western blot in EPI. Similarly, Wernstedt Asterholm et al. (2014) also noted that the total collagen content measured by western blotting in adipose tissue does not necessarily reflect the pathological fibrotic state. A novel finding of our study was in SAT, where we have shown a significant attenuation of HFD‐induced gene expression of collagen VI and its regulators TGFβ1, CCN2/CTGF, TIMP1, and TIMP3 by each exercise program. However, this was discordant with the protein levels of collagen VI where we were unable to demonstrate a significant difference in collagen by PSR staining or IHC staining of SAT. In chow‐fed mice, we observed no significant difference in the effect of the exercise programs on ECM remodeling which was similar to the study done by Kawanishi et al. (2013) showing no effect of END on collagen accumulation and ECM remodeling markers in EPI. However, in BAT, there were no significant changes in the ECM markers with both HFD and exercise interventions, which indicates that ECM remodeling activity in BAT may be minimal.

Proteolysis of extracellular matrix plays a key role in adipose tissue expansion and includes changes in the activities of matrix metalloproteinases (MMPs) and tissue inhibitors of MMPs (TIMPs) (Huber et al., 2007; Meissburger et al., 2011; Minematsu et al., 2012; Qiu et al., 2010). The nonsignificant change in collagen VI protein in the SAT of HFD‐fed mice despite higher mRNA for collagen VI and its regulators TGFβ1, CCN2/CTGF, TIMP1, and TIMP3 suggests that the higher collagen synthesis may have been compensated by increased collagen degradation. These changes in ECM remodeling may indeed be beneficial for SAT expansion but not EPI where restrictive changes such as the increased collagen accumulation were observed. The decrease in collagen VI mRNA coupled with TGFβ1, CCN2/CTGF, TIMP1, and TIMP3 mRNA in SAT and EPI in HFD‐fed exercising mice suggests that exercise may have a protective effect by downregulating the profibrotic markers and decreasing collagen accumulation.

Another novel finding in our study was that both END and HIIT prevented the HFD‐induced inflammatory changes in SAT, EPI, and BAT, which we believe has not been previously reported in the literature across all three fat depots for both forms of exercise training. Both exercise programs prevented the HFD‐induced increases in TNFα and MCP1 mRNA in SAT, EPI and BAT, but a consistent change in CD45 protein was only seen in SAT. The preventive effect on inflammation markers was similar to those seen in a study done by Kawanishi, Mizokami, et al. (2013) in mice and in humans undergoing similar exercise programs with END alone (Bruun et al., 2006). It was however, contrary to the study done by Gollisch et al. (2009) where a lower intensity of exercise occurred because it used a voluntary wheel exercise program to attenuate inflammation in obese mice. The reduction in inflammation with both of the exercise programs in our study was associated with the improvement in insulin sensitivity, which further substantiates the role of adipose tissue inflammation in driving metabolic disease progression during obesity (Davis et al., 2011; Guo et al., 2015). Further, upregulation of the inflammatory markers in EPI rather than in SAT of HFD‐fed mice indicates that visceral obesity may be more prone to an inflammatory state (Sam et al., 2009). Thus regular exercise has an important function to prevent HFD‐induced inflammatory change in adipose tissue. In contrast to HFD‐fed mice, in chow‐fed mice we observed different effects for each of the types of exercise on the change in the inflammatory markers in SAT and EPI. Similarly, in other studies also END exercise failed to produce a change in inflammatory response in EPI (Kawanishi, Mizokami, et al., 2013; Linden et al., 2014).

In contrast to our hypothesis, the role of HIIT exercise was not superior in bringing about metabolically favorable changes: reducing body weight, reducing total fat mass, down regulating adipogenic markers, decreasing ECM remodeling, and inflammatory makers, coupled with a relative increase in lean mass and in the thermogenic markers in adipose tissue. Both END and HIIT exercise did not show any significant effect in chow‐fed mice. But, in HFD mice, both exercise programs were equally beneficial in preventing the deterioration of metabolic health caused by the HFD in WAT. Interestingly, in BAT the effect of both exercise programs was minimal in either chow‐ or HFD‐fed mice. The current study was designed for concurrent exercise starting with the onset of the HFD and development of obesity in mice. It does not answer the question as to whether exercise would be as effective, in an established obese HFD model.

The results in the current work provide a comprehensive description of the changes in three fat depots in response to exercise during the exposure to a HFD. The highlighted findings in this study confirm that exercise can prevent the onset of the metabolic derangements caused by a HFD, when such training is commenced together with high‐fat intake. It also shows that there were different responses in differing fat depots to exercise, with overall benefit seen by both END and HIIT regimens. The extent to which this data from mice can be related to the human situation remains to be confirmed in what would be challenging yet important studies to undertake in individuals on suboptimal diets of saturated fat with caloric excess, with parallel exercise training commencement.

## CONFLICT OF INTEREST

There are no conflicts of interest for any of the authors related to this manuscript.

## AUTHOR CONTRIBUTIONS

Babu Maharjan and Paul Williams were responsible for the concepts and for the original experimental design, and production of the manuscript. Babu Maharjan and Sergio Mattinez‐Huenchullan were responsible for the exercise programs and Babu for the major part of the conduct of the laboratory investigations with help from Sergio. Advice and guidance on assays procedures and the processing of the data produced was provided by Stephen Twigg, Susan Mclennan, and Paul Williams who also reviewed versions of the manuscripts produced by Babu and Paul.

## Supporting information



Supplementary Material
