# Supplementary material for: Exercise induces favorable metabolic changes in white adipose tissue preventing high‐fat diet obesity
Source: Physiol Rep. 2021 Aug 17;9(16):e14929. doi: 10.14814/phy2.14929 (PMC8371352; doi:10.14814/phy2.14929)
Supplement: Supplementary file 1 — Supplementary Material [file PHY2-9-e14929-s001.docx]

## Supplementary methods

## Histology and immunohistochemistry

Harvested adipose tissue for histological studies was placed immediately into 10% neutral buffered formalin for 12 h and transferred to an automated tissue processor (Thermoscientific, Victoria, Australia) where tissue was processed through increasing grades of ethanol and ended in paraffin. The processed tissue was placed into paraffin blocks using an embedding station (Thermoscientific, Massachusetts, USA). The tissue blocks were sectioned at 5µm, de-waxed and rehydrated using standard procedures viz: 2 changes of xylene for 10 min, 2 changes of 100% ethanol for 2min, 2 changes of 95% for 2 min, 70% ethanol for 2 min and then rinsed under running water for 10 min. Haematoxylin staining of slides was done by dipping a haematoxylin bath for 2 min, followed by 3-5 dips in acid alcohol and then blueing in Scotts water for 30 sec. Slides were counter stained with 2 changes of eosin for 40 sec (1).

For Immunohistochemical staining of sections, antigen was retrieved by heating slides in a microwave oven in Tris-EDTA buffer pH 9.0 for 10-15 min. Slides were washed for 5 min in TBST 2 times, treated with 3% hydrogen peroxide for 10 min and rinsed for 5 min in TBST 2 times. Tissue sections were blocked for 30 min with 10% normal goat serum (NGS) diluted in TBST. After draining 10% NGS, primary antibody for collagen VI (1:500, catalog number ab6588, Abcam, Massachusetts, USA) was added and incubated overnight at 4^0^C. Primary antibody was removed and slides were rinsed for 5 min 3 times in TBST. Tissues were incubated with secondary antibody (1:200, Biotinylated anti-rabbit IgG, catalog number BA-1000; Vector Laboratories^@^, Burlingame, CA, USA) at room temperature for 60 min. After rinsing the slides for 5 min in TBST 3 times, tissues were incubated in Avidin-Biotin Complex (Vectastain ABC kit, catalog number PK-4000; Vector Laboratories^@^). Tissue was washed for 5 min in TBST 3 times and incubated in Diaminobenzidine (DAB) (catalog number K3468; Dako^®^, California, USA) for between 20 secs and 5 min depending on the abundance of target protein. Tissue slides were rinsed in distilled water to stop the DAB reaction. Counter staining with haematoxylin as done for 2 min, followed by 3-5 dips in acid and finally blueing for 30 sec. After staining, tissues were dehydrated with 70% ethanol, followed by 2 changes in 95% ethanol and 2 changes in 100% ethanol for 2 min. Tissue sections were dried and mounted with DPX.

**References:**

1. **Harris HF**. On the rapid conversion of haematoxylin into haematin staining reactions. *Journal of Applied Microscopy and Laboratory Methods* 3: 777, 1900.
